# Endometriosis-associated infertility: From pathophysiology to tailored treatment

**DOI:** 10.3389/fendo.2022.1020827

**Published:** 2022-10-26

**Authors:** Giulia Bonavina, Hugh S. Taylor

**Affiliations:** Department of Obstetrics, Gynecology and Reproductive Sciences, Yale School of Medicine, New Haven, CT, United States

**Keywords:** endometriosis, infertility, pathogenesis, ovarian reserve, endometrial receptivity, *in-vitro* fertilization (IVF), stem cell

## Abstract

Despite the clinically recognized association between endometriosis and infertility, the mechanisms implicated in endometriosis-associated infertility are not fully understood. Endometriosis is a multifactorial and systemic disease that has pleiotropic direct and indirect effects on reproduction. A complex interaction between endometriosis subtype, pain, inflammation, altered pelvic anatomy, adhesions, disrupted ovarian reserve/function, and compromised endometrial receptivity as well as systemic effects of the disease define endometriosis-associated infertility. The population of infertile women with endometriosis is heterogeneous, and diverse patients’ phenotypes can be observed in the clinical setting, thus making difficult to establish a precise diagnosis and a single mechanism of endometriosis related infertility. Moreover, clinical management of infertility associated with endometriosis can be challenging due to this heterogeneity. Innovative non-invasive diagnostic tools are on the horizon that may allow us to target the specific dysfunctional alteration in the reproduction process. Currently the treatment should be individualized according to the clinical situation and to the suspected level of impairment. Here we review the etiology of endometriosis related infertility as well as current treatment options, including the roles of surgery and assisted reproductive technologies.

## Background

Endometriosis is a complex and systemic clinical syndrome that can negatively impact on women’s reproductive health and quality of life (1). Chronic inflammation and hormonal dependance are the main underlying pathophysiologic mechanisms that drive endometriosis, and the association of these two key biological features make the natural history of this disease distinct.

A possible relationship between endometriosis and infertility was first suggested in the *Corpus Hippocraticum*, as women suffering from dysmenorrhea were urged to conceive as quickly as possible to increase the chance of become pregnant (2). Today, nearly 10% of women in their reproductive age suffer from endometriosis and about one third of them experience infertility, almost twice the rate observed among women without the disease (3). Up to 50% of infertile women are found to suffer from endometriosis (4).

Despite the clinically recognized association between endometriosis and infertility, the mechanisms implicated in endometriosis-associated infertility are unclear and this condition is currently considered multifactorial. In addition, the diagnosis of endometriosis is currently underestimated due to the almost exclusive reliance on surgical findings, which delays diagnosis until symptoms require surgical intervention. The ability to identify endometriosis also critically depend on surgeon’s expertise and may preclude early recognition and treatment. The average time to diagnostics ranges from 4 to 11 years is reported in these patients, and this delay has a significant impact on health-care utilization and costs (5, 6). Indeed, the absence of macroscopic lesions or clinical features does not exclude the diagnosis of endometriosis, as infertility is often the only health concern. Furthermore, only one-half of women with endometriosis-associated infertility show typical lesions (7). In women with infertility, an early diagnosis of endometriosis is crucial from the perspective of fertility because the burden of the disease could be even more deleterious when compounded by the effect of increasing age on ovarian reserve.

The focus of this review is to provide an update of pathophysiology of endometriosis-associated infertility. We will also discuss current medical and surgical strategies, and the role of fertility preservation and of assisted reproductive technologies (ART) in patients with endometriosis.

## Pathogenesis of endometriosis 

**Figure 1:**
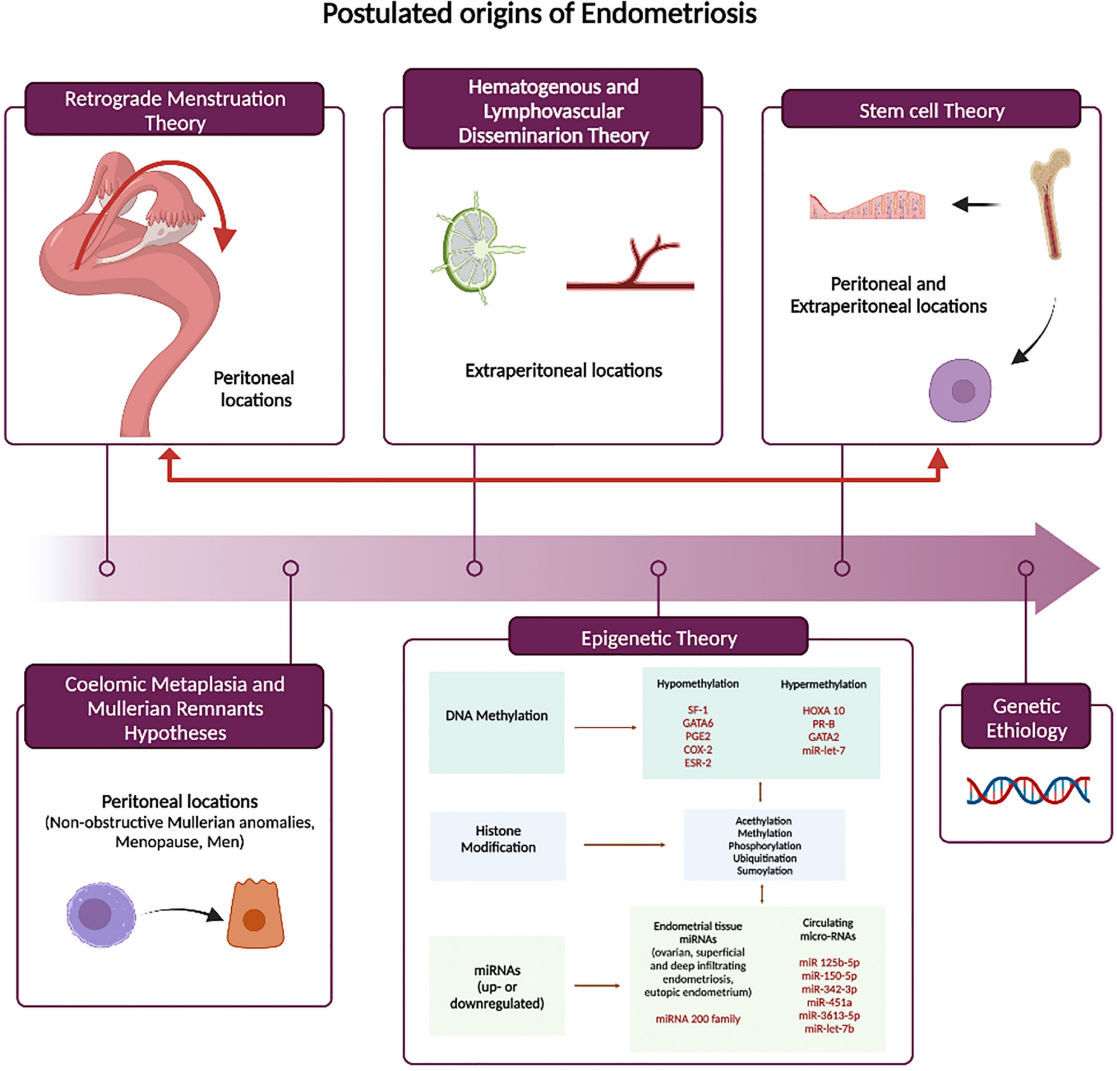
Theories of Endometriosis Pathogenesis. SF-1, steroidogenic factor 1;GATA6, GATA binding protein 6;PGE2, Prostaglandin E_2_;COX2, cyclooxygenase-2;ESR-2, estrogen receptor alpha; HOXA10, homebox protein A10;PR-B, progesterone receptor isoform B; GATA2, GATA binding protein 2.

Understanding the pathogenesis of endometriosis is crucial as it may have meaningful clinical and therapeutical implications. To date, none of the proposed theories have been able to comprehensively explain the natural history of the disease and its associated diverse clinical presentations. The common thread to all theories is a complex dysregulated hormonal signaling, enhanced proinflammatory microenvironment that has the potential to drive the initiation, maintenance, and progression of the disease (**Figure 1**).

### Retrograde menstruation theory

The most widely accepted pathogenetic hypothesis is based on retrograde menstruation as proposed by Sampson in 1927 (8–10). Viable endometrial tissue moves into the pelvic cavity through the fallopian tubes at the time of menses, adheres to the peritoneal mesothelial cells, proliferates, and finally invades pelvic structures. Retrograde menstruation is a physiological event that occurs in approximately 90% of women (11–13). Viable endometrial tissue has been identified in the shed menstrual endometrium (13). However, the differences in its morpho-histological, hormonal, and biological composition compared with the eutopic endometrium of healthy women still remain matter of investigation. Endometrial reflux seems to be enhanced in women with endometriosis and possibly driven by the action of prostaglandins that may cause disorganized myometrial contraction (14–16). Moreover, the incidence of endometrial reflux is much higher in women with congenital anomalies causing menstrual outflow obstruction (17). This theory has been well supported by animal models of endometriosis. Normal endometrial tissue placed into the peritoneal cavity recapitulates the disease, including the effects on eutopic endometrium, suggesting that an abnormal endometrium is not a prerequisite for initiation and development of endometriosis (18–21).

Early age at menarche, long duration and heavy menstrual flow are all well-recognized epidemiological risk factors for the development of endometriosis. The anatomical predominance of endometriosis in the right side of both hemipelvis and diaphragm, further supports this theory (22). This asymmetry has been attributed to both a physiological process (the clockwise intraperitoneal current) and an anatomical factor (the presence of the sigmoid colon and falciform ligament). However, the retrograde menstruation hypothesis is not sufficient to explain the development of rare forms of the disease.

### Coelomic metaplasia and mullerian remnants hypotheses

The coelomic metaplasia and mullerian remnants hypothesis are both based on the concept that endometriotic lesions originate *in-situ* from embryological remnants or by metaplasia. Based on the mullerian remnants hypothesis (“mullerianosis”) (23), endometriosis is a consequence of the aberrant migration and differentiation of embryonic cell rests originating from the Mullerian ducts during organogenesis. This hypothesis can explain the presence of endometriosis in in adolescents before or shortly after menarche and in fetuses (24–26). Embryological studies (24) support the presence of Mullerian remnants in the cul-de-sac area, uterosacral ligaments, and medial broad ligaments. Alternatively, both germinal ovarian epithelium and peritoneum may undergo a Mullerian metaplasia and differentiate into endometrium (27). This latter theory would explain the presence of endometriosis in ovary, sigmoid colon, appendix, or more distal sites such as the diaphragm and pleura (28), although direct infiltration through diaphragmatic fenestrations is possible. Additionally, both hypotheses may explain rare cases of endometriosis in women with Mayer-Rokitansky-Kuster-Hauser syndrome and other non-obstructive Mullerian anomalies (29–31), in the absence of menstruation (menopause) (32) and in men (33–36).

### Hematogenous and lymphovascular dissemination

Sampson recognized that retrograde menstruation does not explain uncommon extraperitoneal locations and diverse clinical presentations with symptoms remote form the pelvis (37). He first suggested hematogenous or lymphatic dissemination of endometrial like-tissue as an alternative theory. This hypothesis implies that endometrial cells enter the uterine vasculature or lymphatic system at menstruation, and they spread to ectopic sites (38). In murine models of surgically induced endometriosis, endometriosis-derived cells are capable of migration and micrometastasis to different extra-pelvic organs including lung, spleen, liver and brain (39). Clinically, this theory has been supported by the presence of endometrial tissue in the uterine vasculature (37) and by evidence of emboli in sentinel lymph nodes (40).

### Stem cell theory

In the last few years, it has become clear that altered stem cell trafficking contribute to the etiology and pathophysiology of endometriosis. The first evidence on the contribution of bone marrow derived stem cells (BMDCs) in the regeneration of the endometrium was reported in 2004 (41); subsequent studies have confirmed the bone marrow contribution to endometrium (42, 43). Both progenitor cells within the endometrium and multipotent cells from bone marrow contribute to endometrial homeostasis. The BMDSCs travel through the circulatory system and contribute to the composition of eutopic endometrium (41). After travelling to endometrium these BMDSCs can become restricted to an endometrial cell lineage, contributing to the pool of both stromal and epithelial endometrial progenitor cells. Some become located in the basal layer of the eutopic endometrium and regenerate on a monthly basis under the influence of estrogens. Furthermore, women with endometriosis have a higher number of these pluripotential cells compared with healthy women during menses (44).

During menstruation, women with endometriosis shed more basalis cells, including progenitor cells, than healthy individuals, these cells can more easily generate endometrium in ectopic locations than differentiated cells and further expand on Sampson’s theory of retrograde menstruation (45).

BMDSCs can directly differentiate into endometrium without first being localized in the uterus. Ectopic differentiation of circulating stem cells has been proposed as a pathogenetic mechanism of endometriosis. Mesenchymal extrauterine stem cells derived from bone marrow and other sources may also be involved in the pathogenesis of the disease both in the peritoneal cavity as well as distant sites. Their inappropriate differentiation to endometrial cells at ectopic locations is likely the principal source of extraperitoneal endometriosis (46, 47).

The ability of BMDSCs to contribute to endometriotic lesion and differentiate into endometrial phenotypes help to explain how ectopic tissue can occur in locations outside the peritoneal cavity and in non-peritoneal-derived cells, such as lungs (48–50), central nervous system (51) and in men (33–36). Furthermore, BMDSC are attracted by eutopic endometrium under injury and inflammatory conditions (52). Endometriotic lesions, through the production and release of pro-inflammatory cytokines and chemokines (47) and under estrogenic influence (53) recruit more stem cells to further promote lesion growth. Additionally circulating endothelial progenitor cells contribute to the vascularization of endometriotic lesions. Stem cells are also capable of trafficking between endometriotic lesions and the eutopic endometrium, and therefore likely contribute to the impaired uterine receptivity in these women (54). These cells, derived from endometriosis, migrate as mesenchymal stem cells (MSC), engraft the uterine stroma, however activation epithelial Wnt signaling that likely distorts the epithelial-stromal dialog needed for optimal endometrial development and receptivity.

### Genetic etiology

Familiar clusters of endometriosis have been found in humans (55) and nonhuman primates (56, 57). However, no distinct inheritance pattern has been established and the notion that multiple genes contribute to endometriosis is widely accepted. Studies on monozygotic twins show that endometriosis has an estimated total heritability of approximately 51% (58–60). Daughters of mothers with surgically confirmed endometriosis have more than double risk of developing the disease (61). Moreover, familial inherited of endometriosis tends to be more severe with an earlier onset of symptoms compared with sporadic cases (55, 62). Meta-analyses of genome-wide association studies of diverse populations have identified a robust association of endometriosis with certain risk loci involved in sex steroid hormone pathways, indicating a possible role in the development of advanced stages of endometriosis (63–68). However, none are common and in total these genetic variants account for only a small fraction disease risk. In general, a multitude of genetic variants with only weak individual effects care likely responsible for the increased hereditary risk of endometriosis (69).

In the context of infertility associated with endometriosis, a recent cross-sectional study including 213 infertile women with endometriosis who underwent IVF procedures, found that single nucleotide variants of FSHB and FSHR separately interfered with the hormonal profile (both FSH and LH levels) and ultimately with the number of oocytes retrieved in these patients at any stage of the disease (70).

### The epigenetic theory

There is a growing body of evidence that epigenetics has a key role in the pathogenesis of endometriosis. Epigenetic modifications involve dynamic and reversible changes in the chromatin structure influencing gene expression in a heritable fashion. Epigenetic phenomena are likely to have implications for diagnosis, prognosis and for the possibility of developing targeted therapeutic strategies. The hallmarks of epigenetic gene regulation are DNA methylation (hypo and hypermethylation), histone modifications, and microRNA production, which lead to expression or suppression of specific proteins. Comparative studies of both ectopic lesions and eutopic endometrium stromal cells have provided data on the role of epigenetic factors in the etiopathogenesis of endometriosis and its related infertility (19, 71–73).

DNA methylation is one of the most common epigenetic modifications and active in endometrium. Numerous studies have revealed a direct correlation with the expression of genes influencing the implantation process in eutopic endometrium of women with endometriosis. Homebox protein-A10 (HOXA10) is a gene that has a well characterized and essential role in generating a receptive endometrium. Hypermethylation of the HOXA gene promoter has been demonstrated both in animals and in the eutopic endometrium of women with endometriosis compared to healthy controls (19, 74, 75). As promoter hypermethylation is generally associated with gene silencing, the reduced HOXA10 gene expression in the endometrium of women with endometriosis is, at least in part, responsible for the impaired uterine receptivity. Conversely, one recent study (76) found hypomethylation of the HOXA10 gene in the endometrium of women with a previous history of endometriosis and under hormonal treatment at the time of surgery, opening the possibility that long-term therapy may reverse epigenetic signatures classically seen in the disease. Aberrant DNA methylation patterns have also been found in endometriotic tissue compared with eutopic endometrium. The promoter of progesterone receptor (PR) isoform B gene is hypermethylated in endometriosis, with subsequent reduced PR-B expression (77–79) contributing to the relatively persistent progesterone resistance. Similarly, the different level of expression and methylation (hypo or hyper) of certain transcriptional factors (GATA6, GATA 2 and steroidogenic factor 1(SF1)) may account for estrogen dependency and progesterone resistance by changing the expression of both estrogen receptor-beta and progesterone receptor (80–82). Lastly, the invasive proprieties of endometriotic cells have also found to be regulated by hypermethylation in endometriosis (83–85). Let-7 microRNA is hypermethylated in endometriosis leading to decreased Let-7 expression and disinhibition of KRAS and other genes that drive endometriosis growth and invasion (86).

Little is known about the role of histone modifications in the pathogenesis of endometriosis, and results are often conflicting. A marked histone hypoacetylation has been shown in endometriotic stromal cells of both eutopic and ectopic tissue of affected women compared to healthy endometrium (87) and HDAC enzymes seems to play a key role in this process (88–91). Also, acetylation levels of H3 and H4 histones are lower in ectopic lesions and eutopic endometrium of women with endometriosis compared with healthy women (92, 93).

MicroRNAs (MiRNAs) are small RNA molecules of approximately 22 bases. They interact with mRNA and change gene expression by inhibiting translation or inducing mRNA degradation. Their increased expression causes repression of translation from the mRNA while decreased MiRNA expression can lead to upregulation of protein production from mRNA. They also target and regulate both methylation and acetylation processes, thereby modifying the epigenome. Unlike other epigenetic mechanisms, miRNAs regulate gene expression at a post-transcriptional level, and they are found both intra- and extracellularly (94). They target genes involved in hormone metabolism, cell cycle proliferation, migration, and invasion, immune- inflammatory response, epithelial-mesenchymal transition (EMT), apoptosis and angiogenesis (95). Differential expression of more than 100 miRNAs has been found in paired endometriotic lesions and eutopic endometrium of women with and without endometriosis (96, 97). In addition, different expression profiles were detected and reported as characteristic to each lesion subtype (ovarian, superficial, and deep infiltrating endometriosis) (98). Moreover, miRNA signatures in endometrium are likely to change with the respect of the different phases of the menstrual cycle (99, 100). The most frequently detected miRNA both in endometriomas and endometriotic lesions, found to be downregulated in six studies was miR-200 family, known to play a crucial role in the EMT, a relevant process in the establishment of endometriotic lesions (101, 102). Other miRNA reported to be differentially regulated (up- or downregulated) in endometriotic lesions in more than two studies were miR-1, -29c, -34c, -100, -141, -145, -183, -196b, -200a, -200b, -200c, -202, -365, and -375 (103). Several of these are also known to be involved in EMT, as well as, cell proliferation, cell adhesion, invasion and angiogenesis and demonstrating binding to target mRNAs is an important step to validate their role in the pathogenesis of the disease. Extracellular miRNAs are found in all body fluids, including the circulation (both serum and plasma) (104). Circulating microRNAs can potentially impact endometriotic lesion development by mediating intercellular communication between eutopic endometrium and ectopic implants (105).

## Pathophysiology of endometriosis associated infertility

### Translational animal models of endometriosis associated infertility

Considering the limited knowledge of endometriosis pathophysiology, research has long focused on finding animal models to study suspected pathogenic mechanisms and to find novel targets for therapy. As with human endometriosis, animal models of endometriosis reveal an impact on fecundity in terms of impaired folliculogenesis, ovulation, fertilization, implantation or embryonic developement (106). Non-human primates have been extensively used as experimental models for endometriosis because of their phylogenetic proximity to humans. They menstruate cyclically and therefore they can develop endometriosis spontaneously, resembling the human disease based on retrograde menstruation. To date, 11 species of menstruating non-human primates have been reported (107). Ectopic lesions are laparoscopically and histologically identical and at a similar pelvic sites (108). However, spontaneous endometriosis develops slowly, at a lower rate compared to humans and might be multifactorial. Therefore, alternative methods of artificially induced endometriosis have been introduced in these species: cervical repositioning (109), cervical occlusion (110) or surgical induction (18, 21). The use of the non-human primate model of endometriosis, either inducible or spontaneous, seems to provide an excellent tool to investigate not only the pathogenesis of disease but also its associated infertility and impaired endometrial function. These models offer the opportunity to investigate the effect of endometriosis on the eutopic endometrium because of their similar reproductive physiology and endometrial pattern compared to humans. However, high costs, restricted facilities and ethical challenges are limiting their use for experimental purposes. Conversely, rodents do not menstruate and therefore they do not develop endometriosis spontaneously. Only homologuos models of surgically induced endometriosis have been used so far in this setting. Heterologous mouse models consisting of immunodeficient mouses do not seem offer obvious advantages in the study of endometriosis associated infertility. Ectopic transplanted tissue grows and behaves in a hormone-dependent manner, and they exhibit similar histological patterns compared with human endometriotic lesions (106). Despite of these limitations, the rodent model offers a low cost option and the opportunity to perform studies on large homogeneous groups of genetically similar animals. Transgenerational and long-term studies can also be performed because there is no rejection of the transplanted ectopic tissue. For an accurate model of the human condition, an intact hypothalamic-pituitary-ovarian axis in the animal recipient is essential for the evaluation of endometriosis and its related infertility. Another challenge is the difficulty in developing models that recapitulate all subtypes of endometriotic disease and therefore individualize and target. For example, there is a lack of specific *in vivo* models which resemble characteristics of ovarian endometriosis and its related infertility. To date, few animal models of ovarian endometriosis have been successfully implemented (108, 111, 112). Spontaneous ovarian endometriosis in non-human primates is also not as common as in humans (108). Lastly, a major limitation of these models is the concomitant establishment of other subtypes of the disease within the peritoneal cavity causing potential confounding effects. To capture the full extent of human disease it is possible to transplant human endometriosis into an immunodeficient mouse. This model may best recapitulate human disease (113).

#### Role of pain

For a successful natural conception, the feasibility of sexual intercourse is an important prerequisite, and one that is often neglected, however this is a potentially relevant mechanism involved in endometriosis-associated infertility. Pain may be a factor involved in endometriosis-related infertility when superficial dyspareunia (pain occurring in or around the vaginal introitus) makes intercourse difficult to achieve or deep dyspareunia makes intercourse difficult to sustain, leading to avoidance to sexual activity. However, only a few studies have focused on the relationship between superficial dyspareunia and endometriosis and is often concomitant with deep dyspareunia (114, 115). One cross-sectional study conducted on 300 women with histologically confirmed endometriosis reported that the severity of superficial dyspareunia was associated with increased odds of infertility concerns (116). Endometriosis is associated with a 9-fold increased risk of deep dyspareunia mostly due to the infiltrative form and severe stages of the disease affecting the posterior vaginal fornix, the pouch of Douglas, the uterosacral ligaments, and the rectum (117–119). Although relatively frequent, dyspareunia, is not the exclusive sexual complaint in women with endometriosis. Systematic reviews have highlighted that about two thirds of women with endometriosis have some form of sexual dysfunction not limited to deep dyspareunia (120–122). Chronic, nonmenstrual pelvic pain associated with the disease might influence sexual life by reducing desire, frequency of sexual intercourse, arousal, or orgasm. This will have a significant negative impact on intimate relationships, emotional well-being, and quality of life in general. In this regard, a holistic approach, rather than just a mechanistic approach, is mandatory given the complex nature of human sexuality.

#### Mechanical factors

Pelvic adhesions and anatomical distortion potentially affect the conception process in endometriosis. Inflammation, fibrosis, adhesions, and surgical sequela are the main pathophysiologic processes involved. Anatomical distortion and mechanical factors may impair oocyte release from the ovary, inhibit tubal ovum pick up or ovum transport, and/or block sperm transfer into the fallopian tube. Interestingly, no term pregnancies occurred in a non-human primate animal model of induced endometriosis when adnexal adhesions were noted on the same side as the ovulation occured (123).

#### Ovarian reserve

The ovary is the most common location of endometriosis. Ovarian reserve is one of the main prognostic factors regarding fertility and is in large part related to a woman’s age. Ovarian reserve is defined as the supply of non-growing, unrecruited primordial follicles (124); currently, a clinical tool that accurately predicts ovarian reserve does not exist. Despite concerns over their role and its specificity in clinical practice, antral follicle count (AFC) and serum anti-Mullerian hormone levels (AMH) are currently the most widely used indices of ovarian function (125). AMH is best used in identifying women who may be poor responders to gonadotropin stimulation in the setting of assisted reproductive technologies (ART) (126). AMH concentrations are not greatly affected by the menstrual cycle or oral contraceptives, making measurement possible at any time.

At present, the pathophysiologic mechanism of diminished ovarian reserve in endometriosis remains unclear. Nevertheless, there is a growing molecular, histological, and morphological evidence that endometriomas have a detrimental effect on ovarian function. Whether the endometrioma reduces the amount of functional tissue available by space-occupying effect (mechanical stretching damage) or by a direct toxic effect remains unknown.

An endometrioma is a peculiar benign cyst without a real capsule; therefore, there is exchange of cysts contents with the adjacent healthy ovarian cortex. Unlikely other benign cysts, the fluid of endometriotic cyst is able to induce oxidative stress in viable cells and potentially cause damage to healthy tissue. Molecular comparative analysis of the cystic fluid revealed high concentrations of free iron which is able to mediate the production of reactive oxygen species (ROS) that are highly diffusible through cellular compartments. An increase in the iron concentration in the follicular fluid from follicles in contact with the endometrioma was found in comparison with the contralateral healthy ovary (127). Moreover, proteolytic enzymes, inflammatory and adhesion molecules were also found in the endometriotic cyst fluid microenviroment (128). Thus, the release of toxic cysts contents in the adjacent ovarian parenchyma may lead to oxidative stress, fibrosis, loss of cortical stroma, smooth muscle cell metaplasia, impaired vascularization, and, at later stage, reduced follicular maturation and atresia in early follicles (128). Notably, the demonstration of increased oxidative stress affecting the normal ovarian cortex surrounding an endometrioma strongly suggest a ROS-induced fibrogenic response, leading to inhibition of angiogenesis and to follicular damage (129).

Maneschi et al. (130) first found a reduced follicular number and activity prior to surgery compared in histopathological specimens of endometriomas compared to other benign cysts; these findings were later confirmed in other similar studies evaluating follicular density (131–133). Another histopathological study also found increased fibrotic tissue surrounding endometrioma in comparison with that of other benign cysts (134). Interestingly, focal inflammation in the ovarian cortex of affected ovaries was suggested to cause enhanced follicular recruitment and atresia as a result of fibrosis and loss of cortex-specific stroma that maintains the follicular niche (135). Hence, excessive primordial follicle activation was proposed as a mechanism of “burn-out” of the follicular reservoir in ovarian endometriosis (136). Primordial follicle activation is an irreversible process and results in follicular depletion. The PI3K/PTEN/Akt/FOXO3 and PI3K/Akt/mTOR signaling pathways are the best-characterized regulators of primordial follicle activation during the initial recruitment. Takeuchi et al. (136) demonstrated that the number of primordial follicles was diminished, whereas primary, secondary, antral and growing follicle numbers increased in human ovaries with endometrioma, and this effect was mediated by the PI3K-PTEN-Akt-Foxo3 pathway. Similarly luteinized granulosa cells of women with endometriosis are characterized by increased apoptosis (137), however the specific putative mechanism leading to cell loss has not been yet identified. One recent study (138) found that proteins involved in apoptotic pathways were significantly increased in cortical tissue surrounding small endometriotic cysts (<3cm) but not in those surrounding other benign cysts.

Clinical findings confirm this trend; one recent, large, prospective cohort study including 106,633 premenopausal, laparoscopically confirmed endometriosis patients found higher risk for early natural menopause compared to those without endometriosis, especially in nulliparous women and in those who never used oral contraceptives (139). In two recent meta-analysis both serum AMH and AFC were found to be reduced in patients with unoperated endometriomas compared to patients with other benign ovarian cysts without endometriosis (140, 141). Moreover, in a prospective longitudinal study, a time-dependent effect was recently where serum AMH decline in women with untreated endometriomas faster than in age-matched healthy controls (142). Five meta-analyses (143–146) evaluating reproductive outcomes of women with endometrioma who had not undergone previous adnexal surgery found a reduced responsiveness to ovarian stimulation as measured by higher cycle cancellation rate, lower number of oocytes retrieved and lower number of formed embryos despite similar pregnancy and live birth rates. Besides their overall effect, important questions have been raised concerning endometrioma and the effect of size and bilaterality. Indeed, several comparative studies (147–150) in patients with unilateral endometriomas undergoing IVF showed that the affected and the healthy ovary produce a similar number of codominant follicles and oocytes perhaps indicating more than a local effect in the affected ovary; a single visible endometrioma maybe a marker of bilateral disease or alternatively there may be a systemic effect of the single endometrioma on both ovaries. Women with bilateral endometriomas demonstrate an even lower response to stimulation, however clinical pregnancy rate are not affected (151–153), likely overcome by the availability of multiple eggs and embryos

Another key concern is whether surgery has a negative impact on residual ovarian function. Despite the many studies that have been performed to evaluate the impact of surgical treatment of ovarian endometrioma on ovarian reserve, the data are still inconclusive. The potential detrimental impact of adnexal surgery on ovarian reserve has been elucidated in several histological studies confirming that cystectomy is generally associated with inadvertent removal of healthy ovarian tissue and primordial follicles adjacent to the cyst’s pseudocapsule (154, 155); this effect increases proportionally with cyst diameter (156), and ultimately is poorly correlated with the level of expertise in reproductive surgery (157, 158). Unlike other benign cysts, in which a well-defined capsule is present, endometrioma is not surrounded by a capsule (154) and technical difficulties may arise due to the absence of a clear cleavage plane. However, the damage inflicted by surgery may also be due to the related local inflammation or vascular compromise secondary to excessive manipulation of the cortex with subsequent tearing, bleeding, and the need for electrosurgical coagulation (159). Five meta-analyses showed a significant reduction in serum AMH concentrations after surgical treatment of endometriomas (160–162) and this effect is persistent post-operatively up to 18 months (162) and more pronounced in case of bilateral adnexal surgery (163–165). In contrast, two meta-analyses showed that ovarian reserve evaluated by AFC is not decreased after surgical treatment of endometriomas (162, 166). Concerning reproductive outcomes following IVF treatment, two recent meta-analyses (167, 168) showed a lower number of oocytes retrieved in women who had surgical treatment for endometrioma compared to women with expectant management; this finding was previously confirmed separately in case of unilateral treatment, compared with the contralateral normal ovary without endometrioma in the same patient (144). However, two meta-analyses (144, 169) concluded that women who had surgical treatment before IVF/ICSI had a similar live birth rate, clinical pregnancy rate, miscarriage rate, number of oocytes retrieved, and cancellation rate per cycle compared with those with untreated endometrioma. Lastly, according to a recent cohort study (170), the SAFE (surgery and ART for endometriomas) trial, about 50% of women with endometrioma were able to conceive spontaneously within 6 to 12 months after surgery. On the other hand, higher FSH and LH levels between the 2^nd^ and the 5^th^ day of the cycle prior to IVF required higher doses of gonadotropins for ovarian stimulation, and lower number of oocytes were retrieved after surgery for endometrioma in the remaining cohort of patients addressed for IVF, compared with women with unexplained infertility.

Despite all these efforts, further clinical analysis implementing standardization of endometrioma size, bilaterality, surgical technique, post-operative time-interval evaluations and clinical measurements are needed to help in elucidating both contributions and the magnitude of the effect.

#### Oocyte quality, embryo transport, sperm function and motility, sperm-oocyte interaction

The possible effect of ovarian endometriosis on oocyte quality is still under debate. Deeper understanding of the impact of the disease on oocyte quality is crucial as fertility preservation techniques are gaining attention in the counseling and treatment of this patients. Only few studies have investigated the impact of endometriosis on embryological competence. A recent meta-analysis including 22 studies indicate that endometriosis does not affect embryo morphology: Women with endometriosis have comparable high-quality embryo rate, cleavage rate, and embryo formation rate, regardless the stage of the disease (171). Results from several meta-analyses analyzing IVF outcomes, are controversial due to the high heterogeneity of the included studies (172–175). One recent large cohort study including 3818 embryos in cleavage stage found similar fertilization rate and embryo quality, despite a reduction in viable pregnancy rate. Conversely, one recent retrospective analysis using time-lapse technology observed altered relative kinetics in embryos from patients with endometriosis, supporting poorer embryo quality (176). Lastly, from the oocyte donation perspective, reduced pregnancy and implantation rates are observed when oocytes come from donors with endometriosis (177–179), supporting an effect of endometriosis on embryo quality. In contrast no difference was seen in recipients of donated oocytes based on the presence or absence of endometriosis. However, the cases used in these studies do not reflect the general population of women with endometriosis. The recipients in oocyte donation programs are relatively older compared with the majority of women with endometriosis seeking for pregnancy. With diminishing ovarian reserve and menopause endometriosis typically resolves. A history of endometriosis in a recipient of donor oocytes may not reflect current disease status. Therefore, use of results from oocyte donation does not provide a valid model to evaluate implantation and pregnancy rates in young women with infertility related to endometriosis.

Dysregulation of steroidogenesis and alterations of intrafollicular microenvironment are the main pathophysiological processes investigated in the context of endometriosis. E_2_ is crucial for follicular maturation and oocyte competence; follicular fluid also plays an important role in the reproductive performance of oocytes. Alterations in the normal physiology of the granulosa cells such as increased apoptosis and dysregulations of molecular pathways involved in its development and have been intensively studied. Granulosa cells of women with endometriosis are characterized by a decreased expression of P540 aromatase, a key enzyme in estrogen production. Similarly, some evidence also indicates an altered progesterone secretion from granulosa cells that might affect normal oocyte maturation (180, 181). Symmetrical lower E_2_ levels and higher progesterone levels were found in the follicular fluid of patients with endometriosis compared to controls (182). Moreover, follicular fluid has been shown to be subject to an important oxidative stress (183–188). An imbalance in ROS and antioxidant systems in the oocyte microenvironment could promote abnormal oocyte development, causing DNA damage, which would result in lower oocyte quality. In another study, cryopreserved human oocytes exposed to endometriotic fluid from patients with advanced stages of the disease had excess cellular fragmentation of derived embryos that may lead to impaired embryo development by inducing apoptosis in surrounding blastomeres or by altering blastomere division (189).

An altered systemic and peritoneal immune and inflammatory profile that characterize women with endometriosis has also been proposed to directly influence the follicular fluid composition. Altered levels of pro-inflammatory cytokines and growth factors (IL1B, TNFa, IL2, IL8, IL12, IL6, RANTES) have been reported in the follicular fluid of women with endometriosis compared to controls (190–192). Follicular fluid is released into the peritoneal cavity at each ovulation. Three studies have shown spindle and chromosome damage after incubating murine (193, 194) and bovine (185, 195) oocytes in metaphase II with both peritoneal fluid and follicular fluid derived from infertile women with endometriosis. A reduced implantation rate in normal rabbits was observed when the peritoneal fluid from rabbits with surgically induced endometriosis was transferred (196). On the other hand, intraperitoneal injection of peritoneal fluid from women with endometriosis significantly reduced implantation rates in rabbits as well as in hamsters (197, 198).

Gamete transport is also affected by the inflammatory microenvironment, anatomical distortions and uterotubal dysperistalsis associated with endometriosis (15). The endometriotic pro-inflammatory peritoneal fluid microenvironment may also affect sperm function (199–201) by inducing sperm DNA fragmentation (201), disrupt sperm membrane permeability or integrity (202), reduced sperm mobility (203), impaired sperm-oocyte interaction (204) and abnormal sperm acrosome reaction (205).

#### Impaired ovulation

Clinical data concerning spontaneous ovulation rate in these women is poor and controversial (206, 207). Prolactin levels are significantly higher in women with endometriosis when compared to those of women without endometriosis. Hyperprolactinemia prevents luteinizing hormone pulsatility and interferes with hypothalamic function by blocking estrogen receptors, thus producing anovulation. Another potential cause of ovulation failure in women with endometriosis is the luteinized unruptured follicle syndrome (208), a condition challenging to estimate in clinical settings in which the dominant follicle undergoes luteinization but fails to rupture at or to release the oocyte. Altered patterns of estrogen and progesterone secretion leading to an abnormal luteal phase may also compromise ovulation in these women (209). An association between endometriosis, luteinized unruptured follicle syndrome, and impaired fertility was observed in non-human primates animal models of endometriosis (123, 210) as well as in a mouse model of surgically-induced endometriosis (211, 212).

#### Endometrial receptivity

The implantation rate is clearly diminished in women with endometriosis during both natural cycles and ART treatments, even in patients with minimal disease (213–215). However, data from clinical studies suggesting that endometriosis leads to implantation defects implicating the endometrium is still conflicting (216, 217). Two recent reports showed similar outcomes in terms of implantation rates through ART cycles when compared to healthy controls (218, 219).

Defective implantation could be due to a reduced endometrial receptivity or decidualization capacity in these women.

The eutopic endometrium of women with endometriosis displays several molecular and functional, abnormalities compared to healthy women’s endometrium (220–223). Gradual and profound alterations have also been detected in the endometrium of endometriosis-induced baboons (224, 225). However, the mechanism and specific signal that leads to alterations in the endometrial microenvironment of women with endometriosis is not fully characterized and is still unknown whether changes in the endometrial pattern are the cause for the infertility and for presence of ectopic lesions or vice versa.

Endometrial receptivity and decidualization is dependent upon hormonally regulated molecular processes. Estradiol (E_2_) and progesterone (P_4_) responsive signaling pathways are regulated in an epithelial and stromal compartment-specific manner in the endometrium. Progesterone is the main hormone responsible for the transient receptive endometrial phenotype, essential for embryo implantation. The endometrial response to P_4_ is characterized by inhibition of estrogen-dependent proliferation of epithelial cells, secretory maturation of the glands, and transformation of stromal cells into specialized decidual cells. Functional dysregulation of steroid hormone signaling in endometriosis, such as upregulation of E_2_-induced cell proliferation, inflammation and progesterone resistance, seems to play an important role in impairing endometrial receptivity in these patients (226, 227). The shift toward estrogen dominance promotes inflammation, angiogenesis, cell proliferation, and immunosuppression. Both total endometrial PR expression and PR-A/PR-B isoforms ratios are dysregulated in the endometrium of women with endometriosis (222, 228–230) and in mice with induced endometriosis (19). Moreover, progesterone receptor expression levels are lower in women with endometriosis associated-infertility (231), whereas estrogen receptor 1 (ESR-1) levels are increased in the mid- secretory phase endometrium of these women compared to controls (232, 233).

From a histological perspective, Noyes et al. in 1950 have been proposed eight morphological criteria to evaluate endometrial receptivity, and for many decades they were adopted as the main diagnostic tool for detection of endometrial receptivity defects. However, these criteria have been questioned in recent years and several randomized control trials (RCT) (234, 235) have invalidated their use based on data demonstrating that histological dating of the endometrium does not discriminate between fertile and infertile women. Similarly, the negative predictive value of the endometrial thickness and the endometrial pattern as ultrasonographic parameters (236–239) in predicting endometrial receptivity are insufficient (240).

A transition from an anatomical and histological to a molecular perspective led to the genome-wide screening of all transcribed genes. Transcriptomic analysis of both eutopic and ectopic endometrium from women with or without endometriosis found dysregulations of selected genes that are implicated in the implantation process (241). Interestingly, HOXA10 is a progesterone target in the endometrium. The homebox gene family is critical in the development of the female reproductive tract during embryonic stages as well as in the regulation of endometrial receptivity during adulthood in response to steroid hormones (242). Decreased HOXA10 and HOXA 11 expression has shown to be involved in impaired endometrial receptivity, resulting in decreased implantation rates (75, 243). Patients with endometriosis do not show the normal physiologic rise in HOXA10 and HOXA11 during the mid-luteal phase of the menstrual cycle (75, 243, 244). HOX gene expression is also subjected to epigenetic modifications that leads to long-lasting alterations in endometrial receptivity (245). HOXA10 hypermethylation is an important mechanism responsible for its diminished expression (74). Both murine and baboon endometriosis models showed hypermethylation of the promoter region of HOXA10 and decreased expression of HOXA10 genes in the eutopic endometrium (19, 246). In humans, hypermethylation of HOXA10 was also identified in the endometrium of women with endometriosis (71, 74). Lastly, under normal conditions, high expression of the *HOXA10* gene suppresses the transcription of the EMX2 gene, which is also essential in regulating endometrial receptivity and implantation. With the diminished expression of HOXA10 in endometriosis, the increased level of endometrial EMX2 directly affects endometrial cell proliferation and function during the peri-implantation period, resulting in aberrant implantation (247).

Integrins are cell adhesion molecules expressed in the endometrium during the receptive window and therefore involved in successful implantation. Interestingly, B3-integrin subunit is a direct downstream target gene of both HOXA10 and ESR-1 (248) and its aberrant expression have been described in the endometrium of women with endometriosis. Moreover, in the clinical setting, ART is less effective in patients with lower expression level of B3-integrin in the eutopic endometrium (249). Other transcriptional factors involved in regulation and mediation of progesterone signaling (IGFBP1, GATA2, FOXO1, ARID1A, NOTCH1and WNT4), required for successful implantation are also reduced in endometrium of women with endometriosis. In human endometrial stromal cells, silencing of GATA2, diminishes markers of decidualization (82) and interestingly the expression level is significantly reduced in the endometrium of women with endometriosis (80). Defects in decidual response have been also investigated in the eutopic endometrium of women with endometriosis. Compromised decidualization of cultured stromal cells was found in fresh shed endometrium as well as in the eutopic endometrium of women with endometriosis compared with matched healthy controls (250, 251). Several pathways have found to be aberrant in endometriosis (251–256) contributing to the unfavorable environment and promoting aberrant effects on the maternal/embryo interface. Increased activation of PI3K/AKT (252) pathway and decreased NOTCH signaling (256) contributes to inactivates FOXO1, an important mediator of decidualization involved in the progesterone signaling. Moreover, AKT pathway has been shown to downregulate and upregulate ESR-2 and ESR-1, respectively, with the net effect of promoting estrogen dominance (257, 258). Lastly, IGFBP1, a downstream target gene of HOXA 10 and a marker of decidualization, is reduced in the endometrium of women with endometriosis (251), and it is also downregulated in the eutopic endometrium of mice with induced endometriosis (19).

It is still poorly understood how the immune system contributes to and influences the endometrial microenvironment and the implantation window. Data is conflicting on the immune cell population of both ectopic and eutopic endometrium of women with endometriosis and controls, especially regarding absolute numbers, markers, activation states and cycle dependence due to heterogeneity of studies (259). Eutopic endometrium microenvironment of women with endometriosis seems to be more pro-inflammatory than controls and aberrant functions of certain immune population may lead to an inhospitable environment for embryo implantation. Interestingly, type I classically activated macrophages, that secretes proinflammatory factors, are the main population in eutopic endometrium of women with endometriosis, across all cycle phases, compared with normal controls (260–262). This proinflammatory predominance may decrease embryo nidation. The relative less cytotoxicity of natural killers together with their higher immaturity in the eutopic endometrium of women with endometriosis was significantly correlated with the infertility status in the same group (263). Concerning the role of B cells, anti-endometrial antibodies may also play a role in impaired implantation by affecting directly endometrial function for embryo receptivity (264). Finally, circulating and endometrial/decidual regulatory T cells (Tregs) have shown to be reduced in women with recurrent pregnancy loss, recurrent implantation failure and endometriosis (265).

#### Adenomyosis and other uterine factors

**Figure 2:**
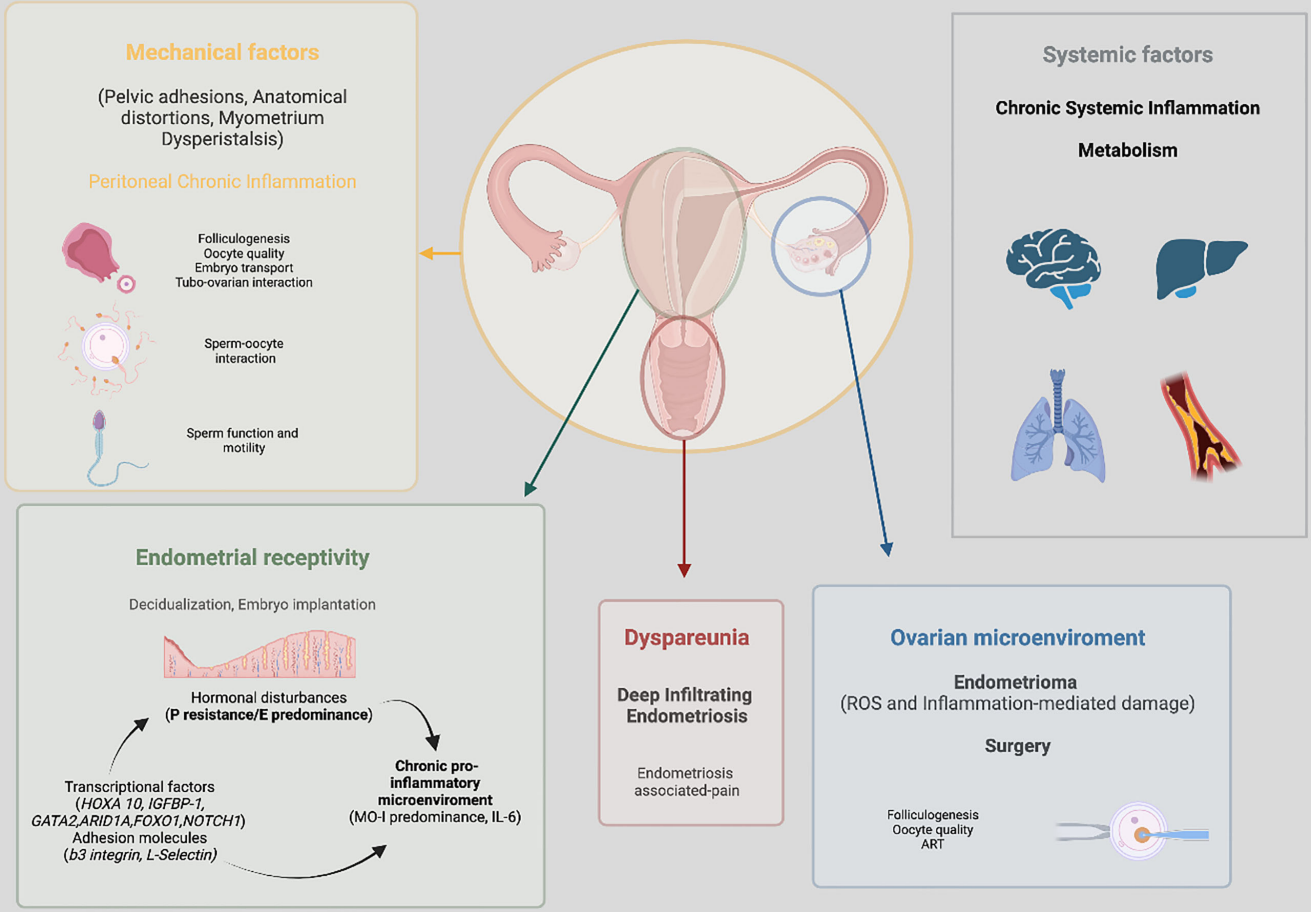
Summary of the main pathophysiological processes implicated in endometriosis associated infertility.

Endometriosis and adenomyosis often co-exist, especially in infertile women (266, 267). Additionally, the concomitant presence of both conditions seems to worsen fertility outcomes (267). In baboons, endometriosis was found to be statistically significantly associated to adenomyosis and the latter was found to be strongly associated with primary infertility (268). Several pathogenetic hypotheses have been postulated regarding adenomyosis and its associated infertility, including junctional zone thickness and subsequent perturbed uterine peristalsis that may alter utero-tubal transport, as well as biochemical, functional and epigenetic alterations in both eutopic and ectopic endometrium (269). The eutopic endometrial microenviroment in adenomyosis differs from the endometrium of unaffected women (270, 271). However, it remains conflicting whether these changes are of clinical significance and, in particular, in the setting of assisted reproductive technologies. According to the most recent meta-analysis in the field (213, 272) the presence of adenomyosis was associated with lower clinical pregnancy rate, higher risk of miscarriage following ART, and (independently of the mode of conception) with adverse pregnancy and neonatal outcomes. However, because of the limited number of comparative studies and their heterogeneous design, the real influence of adenomyosis alone on fertility is still controversial and poorly understood. One recent retrospective cohort study including more than 2000 subjects who underwent ART and by excluding those with decreased ovarian reserve and coexistence of endometriosis and fibroids, found that adenomyosis has a negative effect on IVF outcomes including an increased risk of miscarriage and a reduced live birth rate (273). There are several major limitations in investigating the impact of adenomyosis on infertility. First, there are major diagnostic limitations related with coexistence of endometriosis and adenomyosis, making the interpretation of the available literature difficult. Second, with the advent of 3D ultrasound and the use of magnetic resonance imaging the diagnosis of adenomyosis can now be relatively reliable without the need of histological examination of the surgical specimen (274). However, there is no consensus regarding diagnostic features of adenomyosis using imaging making the interpretation of observational studies challenging; different imaging criteria to define adenomyosis are commonly used. Lastly, adenomyosis frequently coexists with other gynecological disorders and potential confounders, such as uterine leyomiomas. As with adenomyosis, uterine fibroids, in particular submucous leyomiomas, has been associated with lower implantation rates and increased risk for early pregnancy loss (275, 276). The main pathophysiological processes implicated in endometriosis associated infertility are summarized in **Figure 2**.

## Management of endometriosis associated infertility 

Clinical management of infertility associated with endometriosis is challenging due to lack of high-quality scientific evidence and conflicting available guidelines (277). The complexity in therapeutic decision-making is mainly related to the heterogeneous population of infertile women with endometriosis which includes diverse patient’s phenotypes. This often requires innovative diagnostic and therapeutic tools to target the specific dysfunctional step of the reproduction process. Therefore, care of women with endometriosis-associated infertility is best undertaken in referral centers where a multidisciplinary approach can be offered and where both surgery and IVF services are present.

From the patient perspective, a shared and informed decision is mandatory because different treatment options may involve both clinical and personal aspects. The treatment must be individualized according to the clinical situation and to the suspected level of impairment. Factors such as woman’s age, ovarian reserve, duration of infertility, additional infertility factors (male, tubal), ASRM stage, previous surgical treatment for endometriosis, concomitant pain, and indications for IVF-ET must be considered because they will influence the choice of treatment and may also have socio-economic implications.

In American and European guidelines (278, 279), the management of endometriosis is still based on the disease stage defined according to the revised American Society of Reproductive Medicine (rASRM) classification. Despite the high consensus and multiple revisions, the currently used classifications system has several limitations, including failure in predicting fertility outcomes and in accounting for the different types of endometriosis. For this reason, Adamson and Pasta developed a validated and predictive endometriosis staging system, the Endometriosis Fertility Index (EFI), to estimate the non-ART pregnancy rate (natural intercourse or IUI) in women with surgically documented endometriosis (280). This scoring system, which takes into account patient-related factors (age, length of infertility, history of previous pregnancy) and surgical factors (rASRM total score, endometriosis lesions and “least function score” from the tubes and ovaries), is highly accurate (281) and reproducible (282) and represents an important clinical decision tool to counsel patients on their reproductive options after surgery.

While ART can correct many defects that prevent conception, implantation failure is not easily identified, and in most cases, there are no available treatments. The endometrial status is rarely investigated during the standard work-up of infertile women performed in infertility clinics worldwide, even prior to ART (283). Thus, in the era of precision medicine and tailored therapy, a reliable endometrial receptivity assay would be of huge clinical and economical benefit for the patient’s selection process. Starting from functional analysis of the endometrium, Kliman et al. (284) introduced an innovative endometrial functional diagnostic tool (endometrial function test EFT®) based on the use of antibodies two cyclins, as expression patterns of this type of mitotic cycle regulators have been associated with implantation success or failure. Moreover, revolutionary diagnostic tests based on transcriptomic and bioinformatic technologies, that can inform clinicians about the status of endometrial receptivity, have been proposed for diagnostic and prognostic purposes. The endometrial receptivity array (ERA) (285) is a and reproducible (286) microarray-based machine-learning predictive model for assessing endometrial status in the work-up for infertile patients based on the specific signature of 238 differentially expressed genes in the receptive endometrium. The ReceptivaDX™ test a new screening and diagnostic test has been proposed (287) based on the findings that B-cell-lymphoma 6 (BCL6) overexpression in the secretory endometrium of these women contribute to the progesterone resistance (288) and could potentially serve as a surrogate inflammatory marker for a dysfunctional endometrium in endometriosis associated with infertility (289, 290).

Given the important diagnostic delay, strenuous research has been made to identify potential non-invasive diagnostic tool in endometriosis and to date, remains one of the major research priorities in this disease. Several potential biomarkers have been evaluated; however, none has demonstrated sufficient sensitivity and specificity for clinical use.

Cancer antigen-125 (CA-125), a high-molecular-weight glycoprotein antigen expressed in some derivatives of the celomic epithelium, has been previously reported to be elevated in serum of women with advanced forms of the disease (291, 292); however, its overall sensivity is reported to be extremely low (53%) (293).

Based on their pivotal role in the etiopathogenesis of endometriosis, a substantial body of work has shed light on circulating/exosomal miRNAs as potential leading biomarkers for early diagnosis, prognosis, and surveillance for endometriosis. They are they are found both intra- and extracellularly, contained and released *via* exosomes (294).

MiRNAs are attractive due to their simple structure and their stability at the post-translational level and in extracellular biofluids. Conversely, their highly variable content and their low abundance in extracellular biofluids, make detection very demanding. Real time PCR (qRT-PCR) remains the gold standard for miRNA quantification (295). Next-generation sequencing platforms are also used for miRNA sequencing, and they showed high sensivity and excellent reproducibility (296), nevertheless a great performance variation exists among the different platforms. MicroRNA have several targets in cells and each single mRNA transcript may be subjected to regulation by various miRNAs, Thus, the same pathway maybe regulated by a panel of miRNA.

Many studies have investigated the role of circulating miRNA in endometriosis (104, 297–312). To date, more than 60 miRNAs were found to be differentially expressed in the circulation (plasma and/or serum) of women with endometriosis (313). Very few (20%) miRNAs have been replicated in more than one study (314). While generally miRNAs are not highly evolutionarily conserved, serum let-7 family miRNA showed similar dysregulation in a murine model of endometriosis (315).

In general, serum-derived miRNAs seems to yield higher sensitivity and specificity (92 and 95,5% respectively) compared with plasma-derived miRNAs and the highest biomarkers potential was found to be represented by a panel of serum-derived miRNAs comprised of miR-125-5p, miR-150-5P, mir-342-3p, miR-451a, miR-3613-5P, and let-7b (area under the curve (AUC) of 0.94) (313). Cosar et al. (302) reported a logistic regression model combining miR-125-5p, miR-451a, and miR-3613-3p. Later, the same group (300) confirmed a significant diagnostic value of a combination of six miRNAs, with a final AUC>0.9 across two independent clinical data sets.

The reason for the limited consistency of results across studies is related with the dynamic nature/behavior of miRNA expression which is influenced by lack of standardization in the study protocols, such as sample collection (menstrual phase, circadian rhythm), miRNA analysis method, case-control matching and subject’s background (age, ethnicity, health status), stage (minimal-mild vs. moderate/severe) and type of endometriosis (ovarian, peritoneal, deep infiltrating). In addition, different cutoff points were considered to define a meaningful change in expression. An important variable is whether their expression is influenced by the menstrual cycle phase.

**Figure 3:**
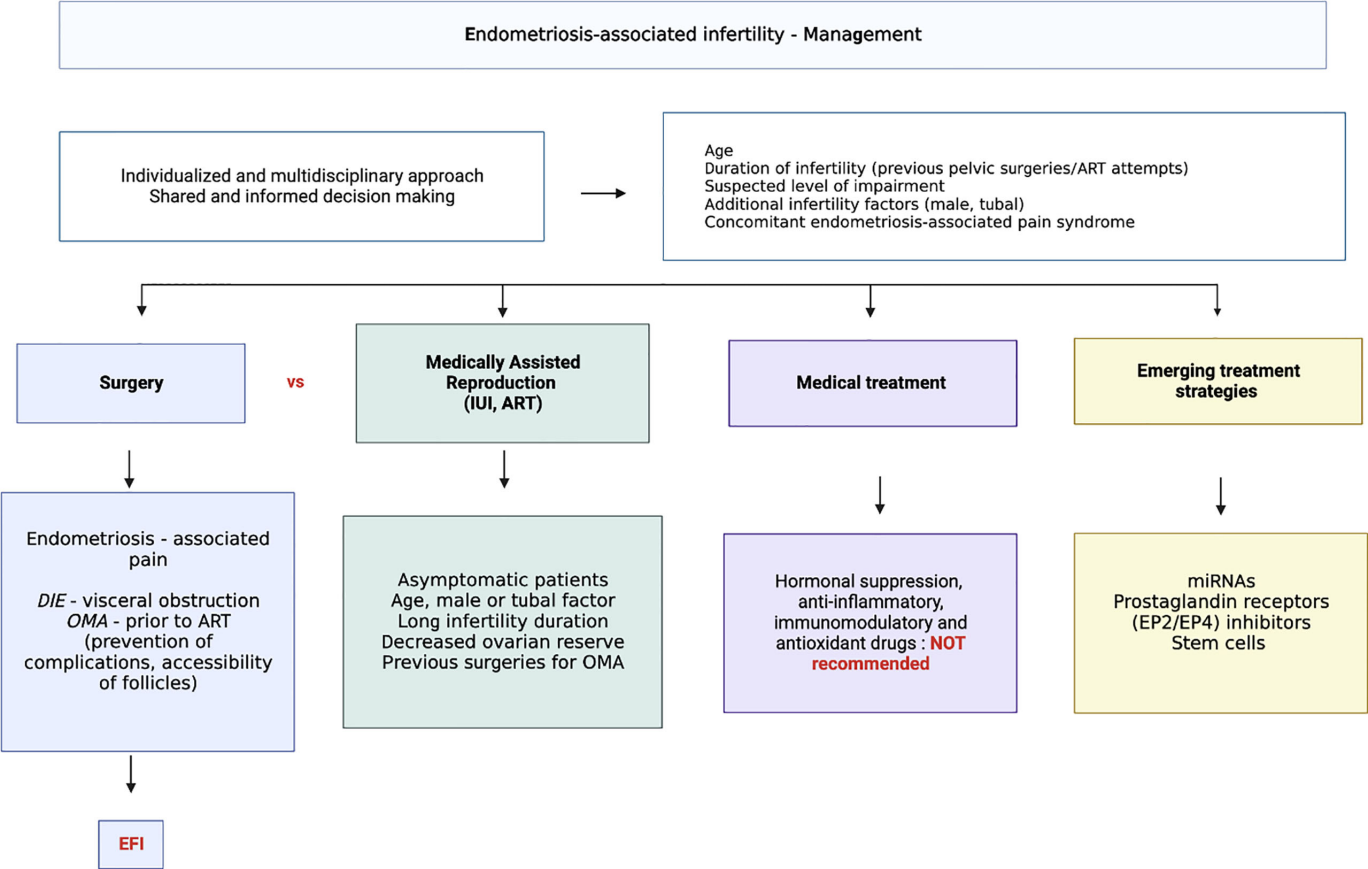
Current and emerging treatment options for endometriosis related infertility.

Results need to be replicated on large series of well-phenotyped patients and under stringent conditions of sampling. The availability of a reliable non-invasive test for endometriosis will allow more accurate and accessible diagnosis as well as the potential for identification and treatment of endometriosis related infertility. Current and emerging treatment options for endometriosis associated infertility are summarized in **Figure 3**.

### Reproductive surgery

Surgical indications should be guided by the presence or absence of pain, patient’s age, history of previous surgery for endometriosis, presence of other infertility factors, ovarian reserve, and estimated EFI. In general, it is clear that multiple surgeries should not be attempted to improve fecundity. Current guidelines suggest fertility counseling before surgery which should include AMH measurement (278, 279).

### r-ASRM stages I and II

Stages I and II are not visible during the clinical and ultrasound examination and they are diagnosed mainly during diagnostic laparoscopy. A recent meta-analysis (315) of moderate quality evidence, including three RCTs on rASRM stage I/II endometriosis, concluded that operative laparoscopy increases natural viable intrauterine pregnancy rates compared to diagnostic laparoscopy only (OR 1.89, 95% CI 1.25-2.86; I^2^ = 0%). Similar findings were later confirmed in a network-metanalysis by Hodgson et al. (316) comparing operative laparoscopy with placebo (OR 1.63; 95% CI 1.13-2.35). Only one meta-analysis (317) analyzed the live birth rate outcome and concluded that laparoscopic surgery in this disease stages has an overall advantage in improving the chances of live birth (RR 1.52, 95% CI 1.26-1.84). Based on this evidence, operative laparoscopy is currently an option for endometriosis-associated infertility in rASRM stage I/II (278, 279) when is performed for other indications such as pain. The absolute benefit is modest with a number of women needed to be treated of 12 to achieve one additional pregnancy.

### r-ASRM stages III and IV

The situation is more complex for advanced disease where high quality evidence regarding the role of surgery for infertile patients is lacking and the risk of major complications due to the surgery itself must be considered. There are no RCT to determine whether clinical pregnancy rates are improved after surgery in patients with stage III-IV of the disease. Women with advanced disease are normally counseled toward surgery in case of significant pain symptoms, large endometriomas, or ureter and bowel clinical involvement.

Apart from the deep infiltrating endometriosis (DIE)-induced alteration of pelvic anatomy and adhesions, evidence supporting a direct link between DIE and infertility is weak and the lack of high-quality data preclude firm conclusions on the effect of surgery. Moreover, DIE alone is found in only 6% of endometriosis patients (318) and surgery-related major complications in this context must be taken into account. According to three independent systematic reviews, pregnancy rate after surgery for rectovaginal endometriosis varies from 24% to 44% (319–321). An additional systematic review (322) of heterogeneous prospective and retrospective studies reported postoperative spontaneous pregnancy rates in women with DIE with and without bowel involvement of approximately 50% and 20%, respectively. Lastly, a recent meta-analysis including observational studies on both rectovaginal and rectosigmoid DIE patients showed a statistically significant benefit of surgery before IVF (323) in terms of live birth and pregnancy rates per patient and per cycle. Nonetheless results need to be interpreted with caution before attributing this rate of success entirely to surgery (324). Interestingly it has been shown in a recent study that extensive surgery in women with deep and intraperitoneal endometriosis, when compared with intraperitoneal surgery only, does not modify the fertility outcome (325). At present, operative laparoscopy for DIE represent a well-established indication in endometriosis associated-pain and in case of visceral obstruction, and is a treatment option in symptomatic patients wishing to conceive (278, 279).

Potential benefits and harms of adnexal surgery for endometrioma must be considered in this context because of its direct effect on ovarian reserve and the risk of recurrence. There is still no consensus regarding the optimal indication for surgery depending on cyst diameter due to the lack of comparative studies. According to guidlines (278, 279), key indications for surgical management of the “asymptomatic” ovarian endometrioma in patients with infertility is to improve the accessibility of follicles and to prevent potential complications (endometrioma rupture, contamination) prior to ART.

A recent meta-analysis (326) conducted by Alborzi et al. of eight prospective studies comparing pregnancy rate from four different approaches of OMA (surgery + ART, surgery + spontaneous pregnancy, aspiration with or without sclerotherapy + ART, and ART alone) found no significant difference between the four study groups. Another meta-analysis (167) analyzing surgical versus expectant management of endometriomas reported similar live birth rates per cycle after IVF in both groups. Surgery is generally not recommended for the sole purpose of enhancing fertility in an otherwise asymptomatic patient.

#### Medically assisted reproduction (IUI, ART)

The utility of Intrauterine Insemination (IUI) with or without ovarian stimulation in patients with endometriosis is supported by only one single RCT (327) including patients undergoing ovarian stimulation with gonadotrophins and IUI versus expectant management. The study found a 5-6 times higher live birth rate per cycle in the treatment group. IUI in combination with controlled ovarian stimulation (clomiphene citrate) is currently recommended only in infertile women with ASRM stage I/II (328).

Currently, up to 25% of *in vitro* fertilization (IVF)-embryo transfer procedures are performed in patients with endometriosis (329). Despite its high implementation, both the influence of endometriosis on pregnancy rates after ART, and the effectiveness of ART treatments in women with endometriosis are still a matter of debate. Main indications for ART remain tubal impairment, male factor, low EFI, and failure of other treatments. There are currently no RCTs evaluating the efficacy of this treatment option versus no intervention in women with endometriosis, and only indirect evidence is available from studies comparing ART outcomes in women with endometriosis to women without the disease. Several meta-analyses (172–175) have investigated ART outcomes of women with and without endometriosis, but results appear conflicting due to the low quality and the high heterogeneity of the selected studies. According to a recent meta-analysis (213), endometriosis consistently leads to reduced oocyte yield and a reduced fertilization rate. Milder forms of endometriosis are most likely to affect fertilization and implantation processes as discussed earlier, whereas advanced stages of the disease may influence all stages of reproduction.

Reasons for the suspected suboptimal performance of ART in endometriosis may include the affected ovarian responsiveness during the ART cycles (low ovarian reserve), impaired endometrial receptivity, and altered folliculogenesis.

Finally, a specific protocol of controlled ovarian stimulation for ART in women with endometriosis is not currently recommended as both antagonist and agonist protocols are still widely used and no difference in pregnancy or live birth rates has already been demonstrated (330).

#### Medical approach

Based on a presumed altered steroidogenesis in endometriosis associated infertility, the use hormonal suppression has been investigated. Based on current recommendations (278, 279) ovarian suppression (danazol, GnRH agonists, progestogens, OCP) should not be offered alone or in combination with surgery in endometriosis-related infertility because there is no evidence of its benefit on pregnancy outcomes (331). A recent Cochrane review (332), comparing the effectiveness of different timing of hormonal suppression in the setting of surgery, concluded that postsurgical medical therapy compared with no treatment or placebo may increase pregnancy rates and reduce disease recurrence, and that it should be recommended in women who cannot, or decide not to conceive immediately after surgery.

The role of downregulation with GnRH agonists prior to ART has been extensively investigated and several meta-analysis have been performed; however results are still contradictory. It has been proposed that medical treatment with gonadotropins prior to IVF may result in improved fertility outcomes in terms of both oocyte quality and endometrial receptivity.

An updated Cochrane review by Georgiu et al. (333) that included 8 RCT concluded that the effect of long-term GnRH agonist pre-treatment for at least 3 months versus no pre-treatment is uncertain in terms of live birth rate (primary outcome), clinical pregnancy rate, multiple pregnancy rate, miscarriage rate, mean number of oocytes and mean number of embryos.

Another more recent meta-analysis (334) investigated the effectiveness of three different down-regulating protocols based on the use of GnRH-agonist (ultra-long, long and short protocol) in infertile women with endometriosis prior to ART. The authors concluded that the ultra-long protocol may improve the clinical pregnancy rate especially in patients with stages III-IV endometriosis based on data from two RCTs. Conversely, more recently, two RCTs (335, 336) failed to demonstrate a beneficial effect of the ultra-long protocol in terms of live birth rate, clinical pregnancy rate, or embryo quality; instead, it was associated with a longer duration of ovarian stimulation, a higher consumption of gonadotropins, and a lower ovarian estradiol production (335). No studies have been conducted to evaluate the efficacy of GnRH antagonists for the treatment of endometriosis-related infertility. A large multicenter RCT (337) is ongoing in the US with the aim to investigate for the first time the pre-IVF treatment with a GnRH antagonist (Elagolix) in women with endometriosis (PREGnant). Compared with GnRH-agonists, the rapid reversibility and recovery of the hormone secretion once the treatment is concluded using GnRH antagonist may allow for better outcomes at the time of ART.

Data addressing pretreatment with continuous oral contraceptives are very limited and do not allow firm conclusion (338, 339). Comparative studies between different hormonal suppression treatment strategies are lacking.

Lastly, assuming that endometriosis is a chronic inflammatory condition, the effect of several anti-inflammatory, immunomodulatory and antioxidant agents has been investigated in the context of inflammation and altered redox balance in the follicular fluid microenvironment and of suspected impaired oocyte quality (340). Pentoxifilline has been the most studied anti-inflammatory and antioxidant agent in endometriosis-associated infertility, and it has also been shown to enhance sperm motility and improve semen parameters in men with oligoasthenospermia. However, according to a recent Cochrane review (341) including 5 RCT, there is no conclusive evidence on its effectiveness and safety in endometriosis-associated-infertility.

#### Emerging treatment strategies

By elucidating the cellular and molecular mechanisms involved in endometriosis-associated infertility and based on the assumption that current hormonal suppression-based treatment cause important side effects rather than effectively improve fertility (342), there is a fundamental need to identify potential signaling pathways for non-hormonal targets for endometriosis associated with infertility.

Non-coding RNAs (ncRNAs) have rapidly emerged as important regulatory molecules in cancer and several reproductive diseases such as recurrent pregnancy loss and endometriosis (95). The use of ncRNAs as a therapeutic tool is still in its infancy; however, the US-FDA has recently approved three RNAi therapies (343, 344). In the context of impaired endometrial receptivity and progesterone resistance, Petracco et al. (345) identified a putative miR135 binding site in HOXA10 gene showing that miR135a and miR135b are expressed in normal endometrium and increased in the endometrium of women with endometriosis; they likely act by regulating targets of progesterone action in the endometrium. Furthermore, miR-451 was found to be the most highly downregulated in the mid-secretory phase of eutopic endometrium of baboons (346) and women with endometriosis (347) compared to controls, leading to an increased expression of transcription factors involved in regulation and mediation of progesterone signaling such as GATA2 and YWHAZ. Similarly, H19 is one of the first long noncoding RNA identified; it is expressed in a menstrual cycle-dependent fashion, confined to the stroma, and acts as a decoy for several tumor-suppressor miRNA. Moreover, it is positively regulated by E2 and negatively regulated by progesterone in the mouse. It was recently found that in the eutopic endometrium of patients with endometriosis, downregulation of H19 will increase let-7 activity, contributing to a decreased proliferation of endometrial stromal cells (through IGFR1 expression inhibition) and contributing to the impaired endometrial preparation and receptivity (through reduction of stromal cell proliferation) (348). Finally, further studies have investigated the role of certain miRNA within progesterone resistance during the luteal phase in women with endometriosis; they reveal that miR-30b, miR-30d, miR-29c and miR-194-3p are up-regulated whereas mi-494 and miR-923 are down regulated in receptive endometrium (230, 349) of both humans and baboons (350). Lastly, upregulation of miR-196 and MEK/ERK signaling proteins was reported in infertile women with minimal/mild endometriosis mediating downregulation of PGR expression and decidualization in eutopic endometrium (351). MicroRNA based therapies are promising new fertility treatments.

In the context of a pro-inflammatory endometrium, one recent study (352) investigated the pharmacological effects of selective inhibition of prostaglandin receptors (EP2/EP4) by using a chimeric mouse model of endometriosis and found that endometrial functional receptivity can potentially be restored the interaction among prostaglandin E_2_ (PGE_2_), estrogens and progesterone. The results indicate that inhibition of EP2/EP4 decreases PGE_2_, estrogen biosynthesis and signaling, pro-inflammatory cytokine production, and increases P_4_ signaling in eutopic endometrium of women with endometriosis.

Stem cell therapy has shown to be promising as a new therapeutic target for infertility, especially Asherman’s syndrome (353). The interaction between BMDSC and endometrial MSC has generated considerable interest because of their tropism toward inflamed foci.

Stem cell properties of self-renewal and differentiation made attractive their use to replace potential damaged tissues and inflammation by reducing intrauterine adhesions and fibrosis (354, 355), improving endometrial thickness (356) and promote endometrial regeneration. To date, the use of stem cell for treating endometriosis, and in particular endometriosis-associated infertility offers an attractive option because of its tropic and immunomodulatory proprieties.

Endometriotic lesions recruit stem cells away from the uterus resulting in inadequate endometrial repair and regeneration as endometriosis more effective in recruiting BMDSCs than eutopic endometrium (357). Lesions highly express CXCL12, a chemoattractant for BMDSCs expressed in many organs, as well as by endometrial stromal cells (357). Inhibiting its receptor (CXCR4)was shown to impact the migration of BMDSCs to the uterus (358). Endometriosis related-chronic inflammation likely acts by continually recruiting BMDSCs to the lesions as demonstrated in animal (52). Moreover, physiologic estradiol levels can increase CXCL12 and CXCR4 expression by endometrial stromal cells and BMDSCs respectively *in vitro*, thereby increasing the chemoattractiveness between the two and consequently, migration (358).

Interestingly, Badoxifene, a selective antagonist ER antagonist, showed to reduce both ectopic endometriotic lesions and the BMDCs engraftment to them, redirecting these cells to the eutopic endometrium (54, 359, 360). This phenomenon might be able to create a new endometrium partially free of epigenetic defects.

The route of stem cell administration will be a crucial component of any stem cell based therapy. Significantly greater levels of stem cell incorporate in uteri of mice when cells were administered systemically by intravenous injection as compared with local injections into the uterus (361). Interestingly, mice that received a systemic infusion of BMDSCs prior to uterine injury were also more likely than twice to achieve a pregnancy, suggesting functional repair of damaged endometrium was due to BMDSs activity (355). BMDSCs maybe superior to ESC in the treatment of uterine injury; they may allow a more complete repair due to their superior versatility, developing into a large number of cell types required for endometrial function (355).

#### Fertility preservation

The need for reproductive counseling utilizing a multidisciplinary medical team has become more evident in endometriosis not only prior to surgery but also at diagnosis, based on the assumption that fertility is likely to be compromised in these women.

Several options are currently available to preserve fertility, including embryo or oocyte cryopreservation and ovarian tissue cryopreservation which are no longer considered experimental procedures (362). Vitrification or planned oocyte cryopreservation technology has grown enormously during the last few years. Several meta-analyses demonstrated that clinical outcomes after vitrification are superior to the standard slow-freezing/thawing. Moreover, comparable results between vitrificated and fresh oocytes were reported (363, 364).

Many questions in terms of efficiency, effectiveness and risks remain unanswered, and the strength of evidence to support fertility preservation in endometriosis, regardless disease severity, is still limited (365). Systematically offering FP to patients with endometriosis might have a dramatic effect on the public healthcare expenditure and may expose patient to unnecessary clinical risks (366, 367). In the context of ovarian endometriosis, subgroups that would particularly benefit from fertility preservation are women with bilateral endometriomas and those scheduled for surgery for contralateral recurrence after unilateral endometrioma surgery or in whom spontaneous conception is unlikely after ovarian surgery (365–368). One of the advantages related with an earlier approach is the opportunity to preserve oocyte at a young age. However, lack of reliable data regarding the effectiveness limit full scale adoption. Women at young age may have a greater risk of recurrence, and when there is not an immediate desire for pregnancy, offering FP could be a beneficial option. Ovarian tissue cryopreservation represents an alternative in case where ovarian hyperstimulation is contraindicated. The first case of autologous ovarian transplantation with cryopreserved tissue was reported in 2000 by Oktay et al. (369). Later updates by the same group in 2010 (370) and Donnez et al. in 2005 (371) reported a similar successful approach in endometriosis cases and to date, other few cases have been reported (372–378).

### Fertility sparing surgery

Optimizing fertility in patients with endometriosis requires reducing potential iatrogenic harm to the ovarian reserve. In this context, the role of adnexal sparing surgery, when indicated, is crucial for treating symptomatic women with endometriosis-associated pain for improving accessibility of follicles prior to ART due to endometriosis-associated infertility. Thus, skilled surgeons with expertise in reproductive pathophysiology are required to avoid potential insults to the healthy parenchyma and ovarian vascular network.

Decrease of normal ovarian cortex (ovarian reserve) and disease recurrence are the two main risks associated with surgery for endometrioma, regardless the surgical technique. Several surgical approaches for endometrioma have been proposed: Excisional, ablative or a combination of both. Lower rates of spontaneous pregnancy and higher rates of recurrence are associated with ablative surgery compared with cystectomy, whereas cystectomy was found to be deleterious for residual ovarian function (162, 379, 380). Since the meta-analysis of Dan et al. in 2013 (380), RCTs comparing ablative versus excisional techniques in terms of ovarian reserve markers and ovarian residual volume have been performed (381–386). Data from animal studies (387) and RCT (381, 382, 388) on the use of laser and plasma energy is encouraging and may result in less inadvertent tissue removal and thermal injury compared with cystectomy and bipolar electrosurgery. Fertility sparing surgery performed in the context of endometriosis-associated infertility also requires the systematic evaluation and optimization of tubal anatomy and patency. Concomitant adhesiolysis with restoration of pelvic anatomy is also recommended when anatomical distortion is present.

## Conclusions

The mechanisms involved in endometriosis-associated infertility are still not completely understood and this condition is multifactorial. Endometriosis-associated pain and inflammation, altered pelvic anatomy and adhesions, disrupted ovarian function, and compromised endometrial receptivity all play a major role in endometriosis infertility in women with endometriosis. Identifying innovative, non-invasive diagnostic tools in endometriosis that also predict a higher risk of infertility remains one of the major research and clinical priorities in this disease; delayed diagnosis allows for disease progression which is clearly detrimental from the perspective of fertility.

Treatment options of infertility associated with endometriosis are still limited. Surgery and ART remain the mainstay of effective therapy. All medical therapies currently approved for use in this disease prevent or diminish fertility and therefore are not helpful in treating this condition. Future non-hormonal medical therapies are needed that can enhance fertility by interfering with the pathways outlined above.

Endometriosis-associated infertility requires a multidisciplinary, personalized, shared and holistic approach based on patient’s unique characteristics, endometriosis subtype and level of impairment.

## Author contributions

GB and HT contributed to manuscript writing and editing. HT revised the manuscript for important intellectual content; all authors approved the final version of the manuscript.

## Conflict of interest

The authors declare that the research was conducted in the absence of any commercial or financial relationships that could be construed as a potential conflict of interest.

## Publisher’s note

All claims expressed in this article are solely those of the authors and do not necessarily represent those of their affiliated organizations, or those of the publisher, the editors and the reviewers. Any product that may be evaluated in this article, or claim that may be made by its manufacturer, is not guaranteed or endorsed by the publisher.
